# ECDC’s latest publications

**DOI:** 10.2807/1560-7917.ES.2018.23.39.1809272

**Published:** 2018-09-27

**Authors:** Eurosurveillance editorial team

**Affiliations:** 1 European Centre for Disease Prevention and Control (ECDC), Stockholm, Sweden

**Keywords:** infectious diseases, public health, Europe

 

**Rapid risk and outbreak assessments**

 

Rapid risk assessment: Monkeypox cases in the UK imported by travellers returning from Nigeria, 2018

Date of publication: 21 September 2018

https://ecdc.europa.eu/en/publications-data/rapid-risk-assessment-monkeypox-cases-uk-imported-travellers-returning-nigeria

 

Rapid risk assessment: Cholera outbreak in Algeria, 2018

Date of publication: 7 September 2018

https://ecdc.europa.eu/en/publications-data/rapid-risk-assessment-cholera-outbreak-algeria-2018

 

Rapid risk assessment: Severe respiratory disease associated with Middle East respiratory syndrome coronavirus (MERS-CoV), 22nd update

Date of publication: 29 August 2018

https://ecdc.europa.eu/en/publications-data/rapid-risk-assessment-severe-respiratory-disease-associated-middle-east-11

 

Rapid risk assessment: Early large increase in West Nile virus infections reported in the EU/EEA and EU neighbouring countries

Date of publication: 13 August 2018

https://ecdc.europa.eu/en/publications-data/rapid-risk-assessment-early-large-increase-west-nile-virus-infections-reported

 

Rapid risk assessment: Ebola virus disease outbreak in North Kivu and Ituri Provinces, Democratic Republic of the Congo

Date of publication: 9 August 2018

https://ecdc.europa.eu/en/publications-data/rapid-risk-assessment-ebola-virus-disease-outbreak-north-kivu-and-ituri-provinces

 

Rapid risk assessment: Public health risks related to communicable diseases during the 2018 Hajj, Saudi Arabia, 19–24 August 2018

Date of publication: 2 August 2018

https://ecdc.europa.eu/en/publications-data/rapid-risk-assessment-public-health-risks-related-communicable-diseases-during-0

 

Rapid outbreak assessment: Multi-country outbreak of Salmonella Agona infections possibly linked to ready-to-eat food

Date of publication: 26 July 2018

https://ecdc.europa.eu/en/publications-data/rapid-outbreak-assessment-multi-country-outbreak-salmonella-agona-infections

 

Rapid risk assessment: Carbapenemase-producing (OXA-48) Klebsiella pneumoniae ST392 in travellers previously hospitalised in Gran Canaria, Spain

Date of publication: 11 July 2018

https://ecdc.europa.eu/en/publications-data/rapid-risk-assessment-carbapenemase-producing-oxa-48-klebsiella-pneumoniae-st392

 

Rapid risk assessment: Dengue outbreak in Réunion, France

Date of publication: 6 July 2018

https://ecdc.europa.eu/en/publications-data/rapid-risk-assessment-dengue-outbreak-reunion-france-0

 

Rapid risk assessment: Multi-country outbreak of *Listeria monocytogenes* serogroup IVb, multi-locus sequence type 6, infections linked to frozen corn and possibly to other frozen vegetables – first update

Date of publication: 3 July 2018

https://ecdc.europa.eu/en/publications-data/rapid-risk-assessment-multi-country-outbreak-listeria-monocytogenes-serogroup-ivb

 

**Scientific advice**

****

Public health guidance on prevention and control of blood-borne viruses in prison settings

Date of publication: 23 July 2018

https://ecdc.europa.eu/en/publications-data/public-health-guidance-prevention-control-bloodborne-viruses-prison-settings

****

**Technical reports**

 

Monitoring the use of whole-genome sequencing in infectious disease surveillance in Europe 2015–2017

Date of publication: 18 September 2018

https://ecdc.europa.eu/en/publications-data/monitoring-use-whole-genome-sequencing-infectious-disease-surveillance-europe

 

Generic protocol on enhanced surveillance for invasive pneumococcal disease at the EU/EEA level

Date of publication: 12 September 2018

https://ecdc.europa.eu/en/publications-data/generic-protocol-enhanced-surveillance-invasive-pneumococcal-disease-eueea-level

 

Hepatitis B and C epidemiology in selected population groups in the EU/EEA

Date of publication: 11 September 2018

https://ecdc.europa.eu/en/publications-data/hepatitis-b-and-c-epidemiology-selected-population-groups-eueea

 

Handbook on tuberculosis laboratory diagnostic methods in the European Union - Updated 2018

Date of publication: 29 August 2018

https://ecdc.europa.eu/en/publications-data/handbook-tuberculosis-laboratory-diagnostic-methods-european-union-updated-2018

 

Synergies in community and institutional public health emergency preparedness for tick-borne diseases in Spain

Date of publication 23 August 2018

https://ecdc.europa.eu/en/publications-data/synergies-community-and-institutional-public-health-emergency-preparedness-tick

 

Synergies in community and institutional public health emergency preparedness for tick-borne diseases in the Netherlands

Date of publication 23 August 2018

https://ecdc.europa.eu/en/publications-data/synergies-community-and-institutional-public-health-emergency-preparedness-tick-0

 

Synergies in community and institutional public health emergency preparedness for tick-borne diseases in Spain and the Netherlands

Date of publication 23 August 2018

https://ecdc.europa.eu/en/publications-data/synergies-community-and-institutional-public-health-emergency-preparedness-tick-1

 

ECDC country preparedness activities, 2013-2017

Date of publication: 21 August 2018

https://ecdc.europa.eu/en/publications-data/ecdc-country-preparedness-activities-2013-2017

 

Fifth external quality assessment scheme for Listeria monocytogenes typing

Date of publication: 21 August 2018

https://ecdc.europa.eu/en/publications-data/fifth-external-quality-assessment-scheme-listeria-monocytogenes-typing

 

Strategy for the external quality assessment of public health microbiology laboratories

Date of publication: 30 July 2018

https://ecdc.europa.eu/en/publications-data/strategy-external-quality-assessment-public-health-microbiology-laboratories

 

ECDC study protocol for genomic-based surveillance of carbapenem-resistant and/or colistin-resistant Enterobacteriaceae at the EU level

Date of publication: 23 July 2018

https://ecdc.europa.eu/en/publications-data/ecdc-study-protocol-genomic-based-surveillance-carbapenem-resistant-andor

 

Systematic review on the prevention and control of blood-borne viruses in prison settings

Date of publication: 23 July 2018

https://ecdc.europa.eu/en/publications-data/systematic-review-prevention-and-control-blood-borne-viruses-prison-settings

 

**Surveillance reports**

 

Monthly measles and rubella monitoring report, September 2018

Date of publication: 14 September 2018

https://ecdc.europa.eu/en/publications-data/monthly-measles-and-rubella-monitoring-report-september-2018

 

Gonococcal antimicrobial susceptibility surveillance in Europe, 2016

Date of publication: 30 August 2018

https://ecdc.europa.eu/en/publications-data/gonococcal-antimicrobial-susceptibility-surveillance-europe-2016

 

Influenza virus characterisation, Summary Europe, July 2018

Date of publication: 21 August 2018

https://ecdc.europa.eu/en/publications-data/influenza-virus-characterisation-summary-europe-july-2018

 

Monthly measles and rubella monitoring report, August 2018

Date of publication: 13 August 2018

https://ecdc.europa.eu/en/publications-data/monthly-measles-and-rubella-monitoring-report-august-2018

 

Influenza virus characterisation, Summary Europe, June 2018

Date of publication: 18 July 2018

https://ecdc.europa.eu/en/publications-data/influenza-virus-characterisation-summary-europe-june-2018

 

Monthly measles and rubella monitoring report, July 2018

Date of publication: 13 July 2018

https://ecdc.europa.eu/en/publications-data/monthly-measles-and-rubella-monitoring-report-july-2018

 

Influenza virus characterisation, Summary Europe, May 2018

Date of publication: 2 July 2018

https://ecdc.europa.eu/en/publications-data/influenza-virus-characterisation-summary-europe-may-2018

 

**Annual Epidemiological Report series on communicable diseases in Europe**

****

Poliomyelitis - Annual Epidemiological Report for 2016

Date of publication: 30 August 2018

https://ecdc.europa.eu/en/publications-data/poliomyelitis-annual-epidemiological-report-2016

 

Tetanus - Annual Epidemiological Report for 2016

Date of publication: 29 August 2018

https://ecdc.europa.eu/en/publications-data/tetanus-annual-epidemiological-report-2016

 

Mumps - Annual Epidemiological Report for 2016

Date of publication: 28 August 2018

https://ecdc.europa.eu/en/publications-data/mumps-annual-epidemiological-report-2016

 

HIV and AIDS - Annual Epidemiological Report for 2016

Date of publication: 21 August 2018

https://ecdc.europa.eu/en/publications-data/hiv-and-aids-annual-epidemiological-report-2016

 

*Haemophilus influenzae* - Annual Epidemiological Report for 2016

Date of publication: 10 August 2018

https://ecdc.europa.eu/en/publications-data/haemophilus-influenzae-annual-epidemiological-report-2016

 

Invasive meningococcal disease - Annual Epidemiological Report for 2016

Date of publication: 9 August 2018

https://ecdc.europa.eu/en/publications-data/invasive-meningococcal-disease-annual-epidemiological-report-2016

****

Invasive pneumococcal disease - Annual Epidemiological Report for 2016

Date of publication: 8 August 2018

https://ecdc.europa.eu/en/publications-data/invasive-pneumococcal-disease-annual-epidemiological-report-2016

 

Legionnaires’ disease - Annual Epidemiological Report for 2016

Date of publication: 8 August 2018

https://ecdc.europa.eu/en/publications-data/legionnaires-disease-annual-epidemiological-report-2016

 

Shiga-toxin/verocytotoxin-producing *Escherichia coli* (STEC/VTEC) infection - Annual Epidemiological Report for 2016

Date of publication: 6 August 2018

https://ecdc.europa.eu/en/publications-data/shiga-toxinverocytotoxin-producing-escherichia-coli-stecvtec-infection-annual

 

Brucellosis - Annual Epidemiological Report for 2016

Date of publication: 6 August 2018

https://ecdc.europa.eu/en/publications-data/brucellosis-annual-epidemiological-report-2016

 

Lymphogranuloma venereum - Annual Epidemiological Report for 2016

Date of publication: 16 July 2018

https://ecdc.europa.eu/en/publications-data/lymphogranuloma-venereum-annual-epidemiological-report-2016

 

Diphtheria - Annual Epidemiological Report for 2016

Date of publication: 16 July 2018

https://ecdc.europa.eu/en/publications-data/diphtheria-annual-epidemiological-report-2016

 

Tick-borne encephalitis - Annual Epidemiological Report for 2016

Date of publication: 16 July 2018

https://ecdc.europa.eu/en/publications-data/tick-borne-encephalitis-annual-epidemiological-report-2016

 

Pertussis - Annual Epidemiological Report for 2016

Date of publication: 16 July 2018

https://ecdc.europa.eu/en/publications-data/pertussis-annual-epidemiological-report-2016

 

Syphilis - Annual Epidemiological Report for 2016

Date of publication: 16 July 2018

https://ecdc.europa.eu/en/publications-data/syphilis-annual-epidemiological-report-2016

 

Gonorrhoea - Annual Epidemiological Report for 2016

Date of publication: 16 July 2018

https://ecdc.europa.eu/en/publications-data/gonorrhoea-annual-epidemiological-report-2016

 

Chlamydia infection - Annual Epidemiological Report for 2016

Date of publication: 16 July 2018

https://ecdc.europa.eu/en/publications-data/chlamydia-infection-annual-epidemiological-report-2016

 

Congenital syphilis - Annual Epidemiological Report for 2016

Date of publication: 16 July 2018

https://ecdc.europa.eu/en/publications-data/congenital-syphilis-annual-epidemiological-report-2016

 

Antimicrobial resistance (EARS-Net) - Annual Epidemiological Report for 2014

Date of publication: 3 July 2018

https://ecdc.europa.eu/en/publications-data/antimicrobial-resistance-ears-net-annual-epidemiological-report-2014

 

Hepatitis B - Annual Epidemiological Report for 2016

Date of publication: 2 July 2018

https://ecdc.europa.eu/en/publications-data/hepatitis-b-annual-epidemiological-report-2016

 

Hepatitis C - Annual Epidemiological Report for 2016

Date of publication: 2 July 2018

https://ecdc.europa.eu/en/publications-data/hepatitis-c-annual-epidemiological-report-2016

 

**Public health guidance**

 

European Union Standards for Tuberculosis Care - 2017 update

Date of publication: 12 September 2018

https://ecdc.europa.eu/en/publications-data/european-union-standards-tuberculosis-care-2017-update

 

**Mission reports**

 

ECDC country visit to Belgium to discuss antimicrobial resistance issues

Date of publication: 11 July 2018

https://ecdc.europa.eu/en/publications-data/ecdc-country-visit-belgium-discuss-antimicrobial-resistance-issues****

**Corporate publications**

****

Achievements, challenges and major outputs 2017: Highlights from the Annual Report of the Director

Date of publication: 29 June 2018

https://ecdc.europa.eu/en/publications-data/achievements-challenges-and-major-outputs-2017-highlights-annual-report-director

 

Annual Report of the Director – 2017

Date of publication: 29 June 2018

https://ecdc.europa.eu/en/publications-data/annual-report-director-2017

****

**ECDC communicable disease threats report**

Date of publication: every Friday

https://ecdc.europa.eu/en/threats-and-outbreaks/reports-and-data/weekly-threats

**

**Scientific peer-reviewed publications**

Publications by ECDC staff in scientific journals

Updated monthly

https://ecdc.europa.eu/en/publications-data?f%5b0%5d=output_types%3A1247

